# Tuberculin Skin Test Result and Risk of Death among Persons with Active TB

**DOI:** 10.1371/journal.pone.0078779

**Published:** 2013-11-11

**Authors:** Sara C. Auld, Eleanor S. Click, Charles M. Heilig, Roque Miramontes, Kevin P. Cain, Gregory P. Bisson, William R. Mac. Kenzie

**Affiliations:** 1 Division of Tuberculosis Elimination, U.S. Centers for Disease Control and Prevention, Atlanta, Georgia, United States of America; 2 Epidemic Intelligence Service, U.S. Centers for Disease Control and Prevention, Atlanta, Georgia, United States of America; 3 U.S. Centers for Disease Control and Prevention, Kisumu, Kenya; 4 Department of Medicine, Infectious Diseases Division, Perelman School of Medicine at the University of Pennsylvania, Philadelphia, Pennsylvania, United States of America; Emory University School of Medicine, United States of America

## Abstract

**Background:**

Although the tuberculin skin test (TST) is frequently used to aid in the diagnosis of tuberculosis (TB) disease and to identify persons with latent TB infection, it is an imperfect test and approximately 10–25% of persons with microbiologically confirmed TB disease have a negative TST. Previous studies have suggested that persons with a negative TST are more likely to present with severe TB disease and have an increased rate of TB-related death.

**Methods:**

We analyzed culture-confirmed TB cases captured in US TB surveillance data from 1993 to 2008 and performed multivariate logistic regression analysis to determine the association between TST result and death.

**Results:**

Of 284,866 cases of TB reported in the US, 58,180 persons were eligible for inclusion in the analysis and 3,270 of those persons died after initiating TB treatment. Persons with a negative TST accounted for only 14% of the eligible cases but accounted for 42% of the deaths. Persons with a TST≥15 mm had 67% lower odds of death than persons with a negative TST (adjusted odds ratio 0.33, 95% confidence interval 0.30–0.36).

**Conclusions:**

A negative TST is associated with an increased risk of death among persons with culture-confirmed TB disease, even after adjustment for HIV status, site of TB disease, sputum smear AFB status, drug susceptibility, age, sex, and origin of birth. In addition to indicating risk of developing disease, the TST may also be a marker for increased risk of death.

## Introduction

Since its introduction in 1890 the tuberculin skin test (TST) has been widely adopted to test for infection with *Mycobacterium tuberculosis* complex (MTBC), both to aid in the diagnosis of tuberculosis disease and to identify individuals with latent tuberculosis infection who might benefit from treatment to prevent progression to TB disease. [1], [2] A positive TST result is based on measurable skin induration in response to intradermally injected purified protein derivative antigens and relies on memory cell immune responses to mycobacterial antigens. However, approximately 10–25% of patients with microbiologically confirmed, active TB disease do not respond to tuberculin and have a negative TST result. [1], [3], [4] Thus, in these individuals the negative TST result does not correspond to microbiologically confirmed *M. tuberculosis* infection.

Previous studies have shown that adults with active TB and a negative TST are more likely to have disseminated disease or pulmonary disease with severe illness. [1], [5], [6] Similarly, young children, people with HIV infection, and people with other immunocompromising conditions such as malnutrition with active TB are more likely to present with disseminated disease and are more likely to have a negative TST when compared with immunocompetent adults. [7]–[9] Children and people with HIV with a negative TST in the setting of active TB have also been shown to have an increased risk of TB-related death in small cohort studies. [10], [11] We are not aware of any studies that report an association between a negative TST and death among other patient populations. However, we hypothesized that other immunocompromised persons, and even persons without overt immune dysfunction, who have a negative TST may also be at greater risk of death from TB disease. Thus, in addition to its diagnostic utility for assessing infection with TB, the TST also may have prognostic value in patients with TB. To further characterize the relationship between TST and TB treatment outcomes, we evaluated whether there is an association between TST result and death among all persons with incident TB disease in the United States over a 15 year period.

## Methods

We analyzed all persons reported as new tuberculosis cases using standardized case report forms to the Centers for Disease Control and Prevention (CDC) National Tuberculosis Surveillance System from January 1, 1993 through December 31, 2008 who had a positive culture result for MTBC at baseline, a documented TST result from the time of diagnosis, and a documented outcome of either completion of TB therapy or death from any cause after initiating TB therapy and prior to completion of therapy. [12] We excluded reports from California because HIV status has not been routinely reported from that jurisdiction. [13] In the interest of comparing groups with similar drug susceptibility patterns and treatment histories we also limited the analysis to persons with (1) drug-susceptible TB (no documented resistance to any of the standard first line drugs [isoniazid, rifampin, pyrazinamide, and ethambutol]) with no prior history of TB who were started on standard four-drug first line therapy; (2) isoniazid monoresistant TB (documented resistance to isoniazid but rifampin-susceptible); or (3) multidrug-resistant (MDR) TB (resistance to at least rifampin and isoniazid). Based on CDC guidelines for the classification of TST reactions, TST result was divided into categories of 0–4 mm, 5–9 mm, 10–14 mm, and ≥15 mm; a TST result of 0–4 mm was considered negative [14].

We compared the proportion of persons who died within each category of TST result using the Pearson’s chi-squared test. We also performed multivariate analysis using logistic regression to determine the association between TST result and mortality. Based on previous data and an a priori interest we adjusted the model for age, sex, and HIV status. [4], [15], [16] We also adjusted for origin of birth (US-born or foreign-born) since most foreign-born persons in the US were vaccinated in their countries of origin with Bacillus Calmette-Guerin (BCG) which can cause a positive TST response. [17], [18] Finally, we attempted to account for severity of TB disease by adjusting the model for anatomic site of disease including the presence or absence of cavitation on chest radiograph for those with exclusively pulmonary disease (i.e., cavitary pulmonary, non-cavitary pulmonary, extrapulmonary only, combined pulmonary and extrapulmonary, and miliary), sputum smear acid fast bacilli (AFB) status at baseline, and the drug susceptibility categories listed above.

### Ethics Statement

Data were collected and analyzed for this project as part of routine disease surveillance activities conducted by the US Centers for Disease Control and Prevention (CDC) and does not constitute research requiring review for the protection of human subjects.

## Results

**Figure 1:**
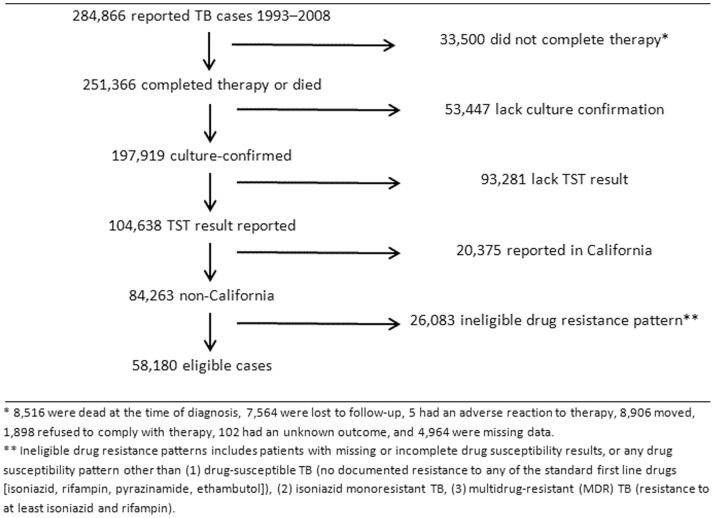
Flow diagram of cases included in the analysis.

From 1993 through 2008, there were 284,866 cases of tuberculosis reported in the United States of which 251,366 either completed therapy or died. There were 8,516 persons who died either prior to or at the time of diagnosis and an additional 20,020 persons who initiated therapy but did not complete treatment for the following reasons: loss to follow-up (7,564), having moved (8,906), adverse reactions (5), refusal to comply with therapy (1,898), unknown reason (15,127). (There were 4,964 patients who were missing data regarding completion of therapy.) Of the cases that completed therapy or died, 197,919 cases had culture-confirmed disease, 104,638 had a documented TST result, and 58,180 met criteria for inclusion in the analysis (Figure 1). People with HIV infection, US-born persons, persons older than 65 years, and those with smear-negative, extrapulmonary, or miliary disease were less likely to have a TST result reported and therefore less likely to be included in the analysis (Table 1).

**Table 1:** Characteristics of all TB cases reported, United States, 1993–2008 and cases excluded or included in the analysis.

Category	All reportedcases N, (%)	% of all reported caseswith a TST result	Cases excluded fromanalysis N, (%)	Cases included inanalysis N, (%)
Total	284,866	56	226,686 (80)	58,180 (20)
Sex				
Male	178,475 (63)	55	140,943 (62)	37,532 (65)
Female	106,339 (37)	57	85,694 (38)	20,645 (35)
Age				
0–4 years	10,518 (4)	86	9,954 (4)	564 (1)
5–14 years	7,361 (3)	88	6,740 (3)	621 (1)
15–24 years	26,328 (9)	71	18,550 (8)	7,778 (13)
25–44 years	100,016 (35)	57	75,892 (34)	24,034 (41)
45–64 years	78,501 (28)	52	61,962 (27)	16,539 (28)
65+ years	61,988 (22)	43	53,360 (24)	8,628 (15)
HIV status				
Positive	29,852 (10)	38	24,830 (11)	5,022 (9)
Negative	104,918 (37)	64	69,196 (30)	35,722 (61)
Unknown	150,096 (53)	54	132,660 (59)	17,436 (30)
Origin of birth				
US-born	152,678 (54)	53	122,139 (54)	30,539 (53)
Foreign-born	130,783 (46)	59	103,238 (46)	27,545 (47)
Site of TB disease				
Cavitary pulmonary	57,183 (21)	58	40,373 (19)	16,810 (30)
Non-cavitary pulmonary	141,474 (52)	59	113,751 (52)	27,723 (49)
Extrapulmonary only	53,217 (19)	52	45,486 (21)	7,731 (14)
Pulmonary/Extrapulmonary	16,878 (6)	50	13,436 (6)	3,442 (6)
Miliary	5,035 (2)	40	4,274 (2)	761 (1)
Smear status at baseline				
Positive	103,984 (37)	52	75,260 (33)	28,724 (49)
Negative	114,317 (40)	62	93,169 (41)	21,148 (36)
Unknown/Not done	66,475 (23)	52	58,181 (26)	8,294 (14)
Deaths	24,472 (9)	31	21,202 (9)	3,270 (6)

Among the 58,180 persons included in the analysis, 14% had a TST of 0–4 mm, 2% had a TST of 5–9 mm, 23% had a TST of 10–14 mm, and 61% had a TST≥15 mm. Of 3,270 deaths, 1,388 (42%) occurred in persons with a negative TST and increasing TST induration size was associated with decreasing odds of death (Table 2). Persons with a TST≥15 mm had 67% lower odds of death than persons with a negative TST (adjusted odds ratio [aOR] 0.33, 95% confidence interval [95% CI] 0.30–0.36). An increased odds of death was found in persons older than 45 years, persons with HIV infection or unknown HIV status, US-born persons, and those with disseminated, AFB smear-positive, or drug-resistant disease. Of note, inclusion of all persons with a documented TST result regardless of culture result (e.g., including cases diagnosed clinically) did not appreciably alter the results of the analysis (n = 145,108∶5–9 mm aOR 0.47, 95% CI 0.40–0.54; 10–14 mm aOR 0.36, 95% CI 0.34–0.39; ≥15 mm aOR 0.28, 95% CI 0.26–0.30).

**Table 2:** Total number of TB cases, number of deaths, and odds of death by clinical and demographic characteristics, United States, 1993–2008.

	TB cases(%)	Deaths(by category)*	Deaths as % ofcases (by row)	Adjusted OddsRatio (aOR)**	95% ConfidenceInterval
Total	58,180	3,270	6		
TST result					
0–4 mm	8,368 (14)	1,388 (42)	17	Ref	
5–9 mm	1,444 (2)	95 (3)	7	0.51	0.41, 0.64
10–14 mm	13,164 (23)	623 (19)	5	0.41	0.36, 0.45
≥15 mm	35,204 (61)	1,164 (36)	3	0.33	0.30, 0.36
Sex					
Male	37,532 (65)	2,314 (71)	6	Ref	
Female	20,645 (35)	956 (29)	5	0.87	0.80, 0.95
Age					
0–4 years	564 (1)	7 (0)	1	0.35	0.16, 0.74
5–14 years	621 (1)	3 (0)	0.5	0.25	0.08, 0.79
15–24 years	7,778 (13)	52 (2)	0.7	0.41	0.30, 0.55
25–44 years	24,034 (41)	757 (23)	3	Ref	
45–64 years	16,539 (28)	993 (30)	6	2.37	2.13, 2.64
65+ years	8,628 (15)	1,454 (44)	17	8.26	7.36, 9.28
HIV status					
Positive	5,022 (9)	796 (24)	16	5.66	5.03, 6.36
Negative	35,722 (61)	1,147 (35)	3	Ref	
Unknown	17,436 (30)	1,327 (41)	8	1.64	1.50, 1.80
Origin of birth					
US-born	30,539 (53)	2,429 (75)	8	Ref	
Foreign-born	27,545 (47)	830 (25)	3	0.55	0.50, 0.60
Site of TB disease					
Cavitary pulmonary	16,810 (30)	739 (24)	4	0.96	0.87, 1.06
Non-cavitary pulmonary	27,723 (49)	1,608 (52)	6	Ref	
Extrapulmonary only	7,731 (14)	371 (12)	5	0.90	0.78, 1.04
Pulmonary/Extrapulmonary	3,442 (6)	280 (9)	8	1.45	1.25, 1.68
Miliary	761 (1)	124 (4)	16	2.36	1.89, 2.96
Sputum smear status at baseline					
Positive	28,724 (49)	1,675 (51)	6	1.42	1.29, 1.56
Negative	21,148 (36)	907 (28)	4	Ref	
Unknown/not done	8,294 (14)	687 (21)	8	1.85	1.64, 2.09
Drug susceptibility class					
Drug-susceptible	52,781 (91)	2,887 (88)	5	Ref	
Isoniazid monoresistant	4,498 (8)	236 (7)	5	1.23	1.06, 1.43
MDR TB	901 (2)	147 (5)	16	5.16	4.17, 6.38

* Pearson’s chi-square p-value <.0001 for all categories.

** adjusted for TST, sex, age, HIV status, origin of birth, anatomic site of disease, cavitation on chest radiograph, sputum smear status at baseline, and drug susceptibility class,

## Conclusions

We observed that patients reported to the US surveillance system with culture-confirmed active TB and a negative TST result were significantly more likely to die after initiation of TB treatment than patients with a positive TST result. This relationship persisted even after adjustment for HIV status, site of TB disease, sputum smear AFB status at baseline, drug susceptibility at baseline, age, sex, and origin of birth.

Our results are consistent with several smaller studies of children, persons with HIV, and immunocompetent adults where a negative TST in the setting of active disease has been associated with a two to three-fold increase in the risk of death. [10], [11], [19] The risk of death associated with a negative TST at the time of disease is also evident in a native Brazilian population with high rates of anergy (46%), severe clinical manifestations, and high mortality from TB disease [20].

The benefit of having a positive TST at the time of disease may have parallels with the effects of BCG vaccination in early childhood. In general, children who have been vaccinated show a transient positive TST response and are less likely to develop disseminated forms of disease, and are less likely to die if they do develop TB disease. [21], [22] Our results suggest that the host immune response in the setting of active TB disease that is measured by a TST might be similar to the immune response triggered by BCG vaccination in children and potentially mitigates against death.

Functional differences have been observed in the cytokine profiles of TB patients who are TST-negative and TST-positive including antigen-specific impairments in IL-2, IL-10, and IFN-γ production. [19], [23]–[25] These impairments in cytokine production and subsequent perturbations in the T-helper 1 and T-helper 2 balance of TST-negative patients may result in impaired ability to control TB disease. Poor clinical outcomes of patients with interferon-gamma receptor mutations and other forms of inherited susceptibility to mycobacterial disease also underscore the importance of cellular immune function, the basis of the TST result, in the successful treatment of TB. [26] Together, these observations indicate that patients with a negative TST may not possess adequate immune function to control TB infection, even in the setting of TB treatment. Future research is warranted to examine how the TST result may serve as an indicator of responsiveness to TB treatment.

Tuberculin skin testing is considered an adjunctive test to support the diagnosis of TB in the United States. [27] Our data indicate that tuberculin skin testing is not consistently performed as only 56% of patients with TB disease in the surveillance database had a TST result reported. We found that persons with HIV, persons older than 65 years, and persons with extrapulmonary or miliary disease were less likely to have a TST result reported. These groups are also more likely to have a negative TST and so it is possible that the association between TST and death is even stronger than what we found in our analysis. We were not able to determine the effect of other immunocompromising conditions on TST and outcome because U.S. surveillance data prior to 2009 do not include information on conditions, other than HIV infection, such as nutritional status, diabetes mellitus, chronic renal disease, or treatments such as TNF-α antagonists that affect immune function and may impact TST results. [1], [4], [9], [28] Furthermore, data from California, which accounts for approximately 20% of TB cases in the United States, were excluded because HIV results were not routinely reported to CDC. Recognizing these limitations of surveillance data it will be necessary to conduct prospective research to verify the findings of this retrospective observational surveillance data analysis.

We recommend increased utilization of the TST in clinical research to systematically evaluate how the TST result may inform clinical care of persons with TB. In addition to serving as a marker of the risk of developing active disease among TB contacts, the TST result appears to be a marker for risk of death in patients with TB disease. However, further research is needed to determine what host or pathogen factors contribute to this increased risk of death observed among patients with a negative TST, and also to determine whether the same associations with death are seen with the newer interferon gamma release assays. Further study of the factors associated with death among patients with a negative TST may help identify strategies to improve survival.
